# Genome wide selection for *Eucalyptus* improvement at international paper in Brazil

**DOI:** 10.1186/1753-6561-5-S7-P44

**Published:** 2011-09-13

**Authors:** Carla Garcia, Bruno Lima, Adriano Almeida, Wesley Marques, Marcos Resende, Roland Vencovsky, Dario Grattapaglia

**Affiliations:** 1 International Paper in Brazil, Mogi Guaçu, SP, Brazil; 2 Department of Genetics, University of São Paulo - ESALQ/USP, Piracicaba, SP, Brazil; 3 International Paper in Brazil; 4 University of Campinas, Campinas, SP, Brazil; 5 EMBRAPA Forestry/UFViçosa, Brazil; 6 Department of Genetics, University of São Paulo - ESALQ/USP, Piracicaba, Brazil; 7 EMBRAPA Genetic Resources and Biotechnology, EPqB 70770-910, Brazilia, DF, Brazil

## Background

The efficiency of plant breeding depends mainly on two actions of the breeder: creation and subsequent identification of superior genotypes. In both actions, selection plays a fundamental role in the definition of crosses to be performed, with interest in creating new genotypes and identifying superior trees to be used commercially. The great attraction of molecular tools for plant breeding is the direct use of DNA information in selection, allowing higher efficiency, quickness in obtaining genetic gains with relatively low costs, when compared to the traditional selection based on phenotypic data. All these objectives can be reached through a new approach: genome-wide selection (GWS) or just genomic selection (GS). Genomic selection can be applied in breeding programs of any species. Results obtained in simulated data indicated that GWS can be very profitable in eucalyptus breeding [1]. GWS is based on selection exclusively by molecular markers, after having their genetic effects estimated based on phenotypic data, in a breeding population sample [2]. The present work aims to characterize and estimate genetic parameters of a hybrid progeny test, the population that will compose the genome-wide selection study at International Paper in Brazil.

## Material and methods

The International Paper Brazil population chosen for genome-wide selection purposes is installed in a Hybrid Progeny Test, comprising 58 crosses from controlled pollination of *Eucalyptus grandis* and *Eucalyptus urophylla* and five common checks (commercial clones of the company), totaling 63 treatments. These 58 families are derived from 56 different parents crossed. The test was installed in July 2006 in Brotas (São Paulo State, Brazil) in randomized complete block design, with six plants per plot and eight blocks, corresponding to a total of 3,024 plants. In 2011 the test was evaluated, performing the measurement of diameter at breast high (DBH) and plant height of the progenies. The obtained data were analyzed in Selegen-REML/BLUP software for the estimation of genetic parameters. The analysis, will allow the identification of the elite individual trees, which comprised the GWS population, ranking the best living trees of the test by estimating the annual average increment and morphological aspects (removing dead, forked and broken trees). Following the selection of the GWS 1,000 trees population, xylem samples were collected and sent for DNA extraction and genotyping with DArT and SNP markers.

## Results

Three variables were analyzed to characterize the test population: DBH, plant height and wood volume (calculated as function of plant diameter and height). DBH had general mean of 13.8 cm and genotypic variance between progenies of 1.61 cmÂ². The individual narrow-sense heritability of the character was 0.255 ± 0.039, very similar to the plant height heritability of 0.236 ± 0.037. Plant height presented general mean of 21.3 meters and genotypic variance of 2.17 mÂ². The estimated wood volume had general mean of 0.168 m3 per plant and genotypic variance of 0.0012 (m3)Â². The individual narrow-sense heritability was slightly higher than for DBH and plant height, namely 0.297 ± 0.042. All estimates were typical of quantitative traits controlled by many loci of small effects. Based on the analysis of the experiment, the best trees were selected, as potential clones and parents for crossing. This analysis revealed 44 crosses with elite clones, to be used as training/discovery population for GWS purposes. The superior individuals of these crosses were selected to complete 1,000 trees, dispersed through the entire test. The number of crosses (44) selected and the different parents identified (45) are important informations for estimating easily the effective population size (Ne), an important factor in GWS [1].

## Conclusions

The information and data generated will lead the studies of the next steps into GWS approach. With the deep understanding of the training population that we have, the genomic breeding values (GBV) can be estimated and the GWS applied using phenotypic and marker data simultaneously. A correct choice of a breeding population is essential for traditional plant breeding and is not different in integrated molecular approaches. Subsequently, the wood phenotyping will be performed and the GBV also estimated for wood quality traits. These data will start up the implementation of GWS in International Paper breeding program in Brazil, as operational procedure, looking at quick selection and, consequently, cost decreases.

**Financial support.** International Paper Brazil, FAPESP, Embrapa-Cenargen
